# A Multiparametric MRI and Baseline-Clinical-Feature-Based Dense Multimodal Fusion Artificial Intelligence (MFAI) Model to Predict Castration-Resistant Prostate Cancer Progression

**DOI:** 10.3390/cancers17091556

**Published:** 2025-05-03

**Authors:** Dianning He, Haoming Zhuang, Ying Ma, Bixuan Xia, Aritrick Chatterjee, Xiaobing Fan, Shouliang Qi, Wei Qian, Zhe Zhang, Jing Liu

**Affiliations:** 1 School of Health Management, China Medical University, Shenyang 110122, China; hedn@cmu.edu.cn; 2 College of Medicine and Biological Information Engineering, Northeastern University, Shenyang 110169, China; 2271371@stu.neu.edu.cn (H.Z.); xuanduth@gmail.com (Y.M.); 2271354@stu.neu.edu.cn (B.X.); qisl@bmie.neu.edu.cn (S.Q.); wqian@bmie.neu.edu.cn (W.Q.); 3 Department of Radiology, University of Chicago, 5841 S Maryland Ave, Chicago, IL 60637, USA; aritrick@uchicago.edu (A.C.); xfan@uchicago.edu (X.F.); 4 Department of Urology, First Hospital of China Medical University, Shenyang 110001, China; 5 Department of Radiology, First Hospital of China Medical University, Shenyang 110001, China

**Keywords:** prostate cancer, castration-resistant prostate cancer, mpMRI, deep learning, radiomics

## Abstract

Objectives: The primary objective of this study was to identify whether patients with prostate cancer (PCa) could progress to denervation-resistant prostate cancer (CRPC) after 12 months of hormone therapy. Methods: A total of 96 PCa patients with baseline clinical data who underwent multiparametric magnetic resonance imaging (MRI) between September 2018 and September 2022 were included in this retrospective study. Patients were classified as progressing or not progressing to CRPC on the basis of their outcome after 12 months of hormone therapy. A dense multimodal fusion artificial intelligence (Dense-MFAI) model was constructed by incorporating a squeeze-and-excitation block and a spatial pyramid pooling layer into a dense convolutional network (DenseNet), as well as integrating the eXtreme Gradient Boosting machine learning algorithm. The accuracy, sensitivity, specificity, positive predictive value, negative predictive value, receiver operating characteristic curves, area under the curve (AUC) and confusion matrices were used as classification performance metrics. Results: The Dense-MFAI model demonstrated an accuracy of 94.2%, with an AUC of 0.945, when predicting the progression of patients with PCa to CRPC after 12 months of hormone therapy. The experimental validation demonstrated that combining radiomics feature mapping with baseline clinical characteristics significantly improved the model’s classification performance, confirming the importance of multimodal data. Conclusions: The Dense-MFAI model proposed in this study has the ability to more accurately predict whether a PCa patient could progress to CRPC. This model can assist urologists in developing the most appropriate treatment plan and prognostic measures.

## Simple Summary

The majority of patients with advanced prostate cancer who receive hormone therapy ultimately progress to castration-resistant prostate cancer (CRPC), which is associated with a significantly poorer prognosis. The current methods to predict CRPC progression rely on invasive and highly time-consuming approaches. This study proposes a multimodal fusion artificial intelligence model that combines multiparametric magnetic resonance imaging data with baseline clinical characteristics of patients to predict whether prostate cancer patients could progress to CRPC after 12 months of hormone therapy. A dense multimodal fusion artificial intelligence (Dense-MFAI) model was developed and validated in this study using data from 96 patients. The model demonstrated a high degree of accuracy in predicting CRPC progression, with a prediction accuracy of 94.2%. This non-invasive approach has the potential to assist urologists in making more informed treatment decisions and developing appropriate prognostic measures for prostate cancer patents, thereby improving patient prognosis through early intervention and personalized treatment strategies.

## 1. Introduction

Prostate cancer (PCa) is the second most common cancer in men worldwide [1]. The prostate cancer screening rate is lower in China than in other developed countries [2]. Consequently, the majority of initial diagnoses occur in the middle or late clinical stages, resulting in a less favorable overall prognosis for prostate cancer patients [3]. Androgen deprivation therapy is currently the primary treatment for men diagnosed with advanced PCa [4]. However, after a median of 12 months of androgen deprivation therapy, almost all advanced PCa patients progress to castration-resistant prostate cancer (CRPC) and the time to progress to CRPC varies considerably between patients [5,6,7]. Once a patient reaches the CRPC stage, the disease rapidly progresses and the prognosis is extremely poor [8].

The clinical progression of CRPC is currently determined by the following three factors: serum testosterone reaching desmoplastic levels (i.e., <50 ng/dL or <1.7 nmol/L), persistent elevation of prostate-specific antigen (PSA) and tumor progression visible in images [9]. However, the use of single indicators such as PSA and serum testosterone for judgments is inherently unreliable [10]. Therefore, there is a pressing need for a method to predict at an early stage whether a patient could progress to CRPC within a specified period following hormone therapy to facilitate clinical decision-making.

Despite significant advancements in precision medicine, proactively and accurately predicting whether a patient could progress to CRPC within a specific time frame remains challenging. A number of studies have predicted PCa progression by fusing multiparametric magnetic resonance imaging (mpMRI) and clinical features. Roest et al. [11] utilized a U-Net model fusing these two modalities to predict clinically significant PCa progression. In contrast to the utilization of a solitary deep learning modeling approach, our study employed a staged and multi-module model design, accompanied by the further integration of information from radiomics. Currently, the majority of methods employed for CRPC prediction utilize biomarkers, the expression of specific genes or proteins and tumor immunohistochemistry [12,13,14]. Jun et al. [15] identified six genes, including NPEPL1 and VWF, to be predictive of CRPC using screening and a regression analysis of 287 genes. Scher et al. [12] demonstrated a strong correlation between the measured circulating tumor cell counts and the progression of CRPC (r = 0.84). However, these methods are associated with high costs, invasive procedures and additional time delays. In contrast, mpMRI—specifically, radiomics feature mapping—and clinical parameters were employed in the present study to construct a multimodal model for prediction. T2-weighted imaging provides anatomical information regarding PCa and dynamic contrast-enhanced MRI (DCE-MRI) aids in the detection of recurrent prostate disease by evaluating the characteristics of the prostate microvascular system [16,17]. Radiomics using tumor heterogeneity features can provide more comprehensive information to accurately predict tumor progression [18,19]. Furthermore, previous studies have demonstrated that baseline clinical features may also provide crucial prognostic information to monitor disease progression in prostate cancer patients [20]. Therefore, a quantitative analysis approach was employed in the present study to predict outcomes, thereby reducing the subjectivity inherent in traditional methods while circumventing the laborious process of the manual testing of genes and proteins that is needed.

Here, we propose an approach based on a multimodal fusion artificial intelligence (MFAI) model to predict whether PCa could progress to CRPC at an earlier time point, at a median of 12 months, on the basis of mpMRI, radiomic feature mapping and baseline clinical data. The implementation of this model by urologists could enable early intervention in high-risk patients and the development of more precise treatment and follow-up plans.

## 2. Materials and Methods

### 2.1. Patients

**Figure 1:**
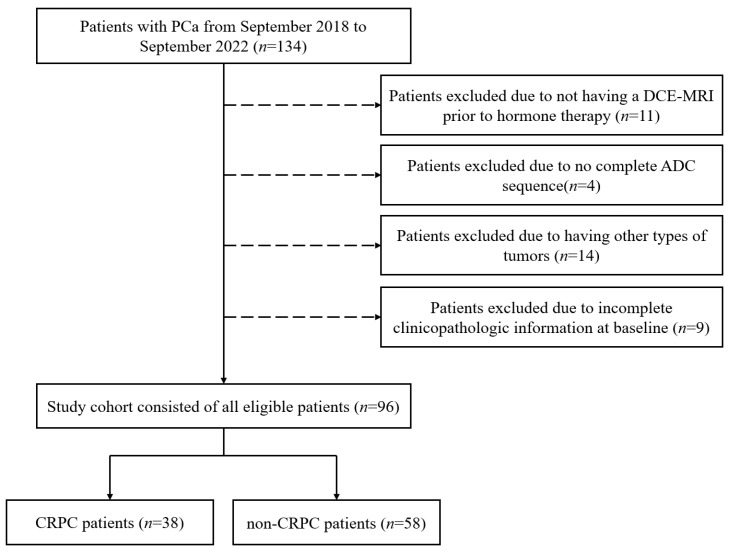
Flowchart of patient selection. PCa: prostate cancer; DCE-MRI: dynamic contrast-enhanced magnetic resonance imaging; ADC: apparent diffusion coefficient; CRPC: castration-resistant prostate cancer.

This retrospective study was approved by the Radiological Review Board of the First Hospital of China Medical University, Shenyang, and adhered to the principles and requirements of the Declaration of Helsinki. No further consent was required for the study. This study involved a retrospective analysis of previously collected data from PCa patients treated between September 2018 and September 2022 at our hospital. The following clinical information was recorded: age, baseline PSA level, Gleason score and clinical TNM stage. The following individuals were excluded from the study: (i) those who did not undergo DCE-MRI prior to hormone therapy and biopsy; (ii) those with an incomplete apparent diffusion coefficient sequence; (iii) those with a history of aggressive malignancy or serious complications in the absence of complete information; (iv) those with other tumor types, confirmed pathologically; (v) those lacking appropriate baseline clinicopathological information; and (vi) those who underwent treatment or tissue biopsy prior to MRI. A total of 96 patients (mean age = 65 years; range 45–88 years) were enrolled in this study; 58 patients did not progress to CRPC after 12 months of hormone therapy and 38 patients progressed to CRPC. The patient recruitment process is shown in Figure 1.

### 2.2. Image Acquisition

MRI was performed using a Siemens VIDA 3T MRI scanner (Siemens Healthineers, Erlangen, Germany). The mpMRI protocols included T2-weighted imaging, diffusion-weighted imaging (DWI) and DCE-MRI. DWI used SPAIR (SPectral Attenuated Inversion Recovery) fat suppression and b-values of 10, 50, 100, 150, 200, 400, 600, 800, 1000 and 1500 s/mm^2^. DCE-MRI data were acquired at a temporal resolution of 7 s. The total amount of gadopentetate dimeglumine injected was calculated on the basis of the patient’s weight (0.2 mmol/kg) and the imaging time was 4.3 min. The specific parameters of the MRI are presented in Table 1.

### 2.3. Data Analysis

The DCE-MRI signal enhancement rate (α), apparent diffusion coefficient (ADC) values and T2 values were calculated on the basis of mpMRI. Previous studies have also demonstrated the effectiveness of these parameters at identifying lesions [21]. Consequently, the three quantitative parameters were selected for the purpose of predicting whether a patient could progress to CRPC.

#### 2.3.1. α-Mapping Acquisition

The percentage signal enhancement (PSE) per pixel in DCE-MRI was calculated as shown in Equation (1), where *S*(*t*) is the signal intensity curve and *S*_0_ is the baseline signal before contrast-agent injection.

(1) $$PSE\left(t\right)=\frac{S\left(t\right)-S_{0}}{S_{0}}\cdot 100$$

Then, an empirical mathematical model was used to fit the *PSE*(*t*) as follows:

(2) $$PSE\left(t\right)=A(1-e^{-\alpha t})e^{-\beta t}$$

where *A* is the amplitude of the *PSE*, *α* is the rate of signal enhancement and *β* is the elution rate. Instead of performing overall averaging directly over the ROI, this process first calculates the percentage signal enhancement profile (*PSE*(*t*)) (Equation (1)) independently for each voxel of the DCE-MRI sequence and then fits the parameter α (Equation (2)) to that voxel based on an empirical mathematical model. Then, we averaged the values for all the voxels in the ROI for our analysis.

#### 2.3.2. T2 Mapping Acquisition

The T2 value was calculated from multi-spin–echo images via a monoexponential signal model according to Equation (3), as follows:

(3) $$S=S_{0}\exp(-\frac{TE}{T2})$$

where *TE* is the echo time, *S* is the signal strength corresponding with different echo times and *S*_0_ is the signal strength at the moment when *TE* = 0.

#### 2.3.3. Apparent Diffusion Coefficient Mapping Acquisition

The apparent diffusion coefficient value was calculated from diffusion-weighted imaging via Equation (4), as follows:

(4) $$S=S_{0}\exp(-b\cdot ADC)$$

where *b* is the diffusion weighting factor, *S*_0_ is the undiffused spin–echo signal and *S* is the diffusion-weighted attenuated spin–echo signal. The *b*-values of this experiment were 10, 50, 100, 150, 200, 400, 600, 800, 1000 and 1500 s/mm^2^.

#### 2.3.4. Long-Run High Gray-Level Emphasis Mapping Acquisition

Our research team previously reported that the long-run high gray-level emphasis (LRHGLE) features calculated on the basis of the gray-level contour matrix in α-parameter mapping are significantly influencing factors for determining sensitive resistance to hormone therapy in prostate cancer [22]. Consequently, we also calculated the radiomic feature mapping. Each pixel was designated as the center of a matrix with a size of 5 × 5, which was used to calculate the LRHGLE features. The average value was then assigned to the center pixel. In this manner, the LRHGLE texture feature mapping was constructed.

### 2.4. Data Preparation

**Figure 2:**
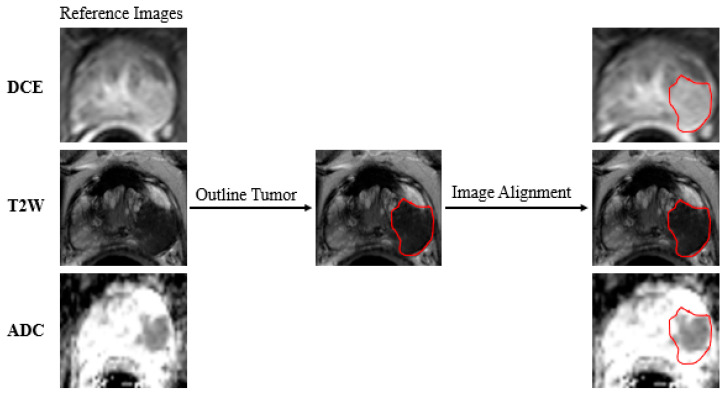
The protocol for tumor marking. The tumor-marking process was executed in T2W images and aligned with DCE and ADC images. T2W: T2-weighted; ADC: apparent diffusion coefficient; DCE: dynamic contrast-enhanced.

**Figure 3:**
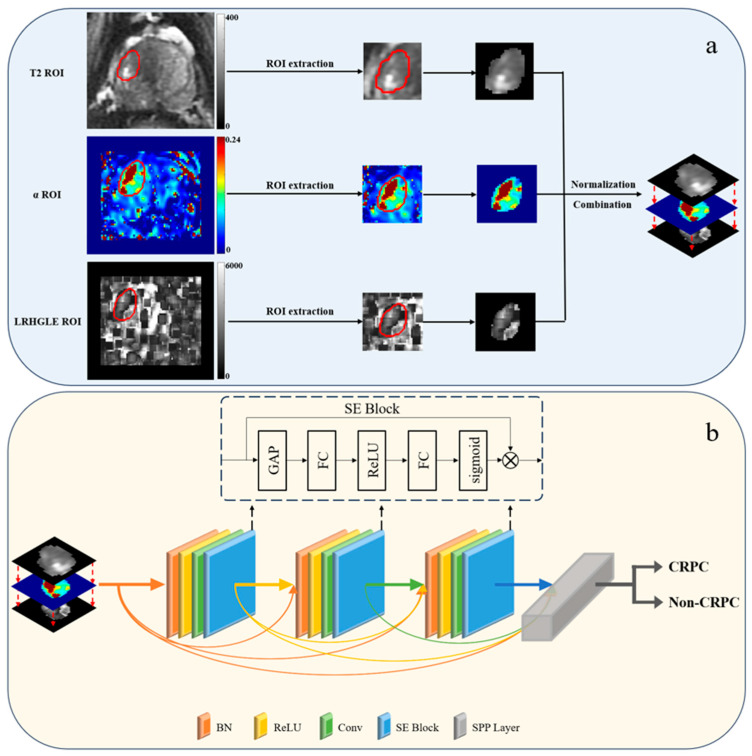
Data preparation process (**a**) and SE-SPP-DenseNet structure (**b**). (**a**) The preparation process included the extraction of ROIs from parametric mapping, normalization and the combination of ROIs. (**b**) The preprocessed images were input into SE-SPP-DenseNet, which uses DenseNet as the network skeleton, and the SE block and SPP layer were added to the model. ROI: region of interest; SE block: squeeze-and-excitation block; SPP layer: spatial pyramid pooling layer; BN: batch normalization; ReLU: rectified linear units; Conv: convolutional layer; GAP: global average pooling; FC: fully connected.

In this study, index lesions were manually and independently marked as regions of interest using a semi-automated method involving two experienced radiologists on the basis of the patient’s diagnostic report. In the event of a Dice similarity coefficient for tumor contours less than 0.80 for two radiologists, the contour was considered to be significantly different, prompting a consultation to reach a consensus. To ensure consistency and reliability, the tumor contours delineated by a radiologist with greater experience were used for all subsequent analyses after a consensus was reached. Neither of the radiologists had access to the pathological reports. The regions of interest (ROIs) were based on the sequence that showed the greatest extent of the cancer. They were mapped using T2WI and transferred to other sequences [23]. The ROIs were subsequently mapped from the T2-weighted image to the ADC mapping and DCE-MRI via image alignment. Despite the demonstrated variability in ROI mapping across different MRI sequences, the relatively fixed position of the prostate and its limited mobility enhanced the reliability of the alignment process [24,25]. Moreover, the accuracy of ROIs has been validated by experienced radiologists following the alignment procedure. The protocol for tumor marking is delineated in Figure 2. Subsequently, following the execution of feature mapping calculations, the ROIs were extracted, normalized and combined using extraction. These were then input into the model. The data preparation process is depicted in Figure 3a.

### 2.5. Deep Learning Model Construction

The deep learning models used in this study were implemented via the PyTorch 1.13.1 framework and Python 3.10 (Python Software Foundation, Fredericksburg, VA, USA) and were trained using a single workstation with an Intel Core i9-13900H processor (Intel, Santa Clara, CA, USA), an NVIDIA 4060 graphics card (NVIDIA, Santa Clara, CA, USA) and 8 GB of random-access memory.

In this study, we first constructed a pure deep learning classification model based on SE-SPP-DenseNet. The dataset was randomly divided into five equal-sized subsets. The dataset for each subset was divided into a training–validation–test set ratio of 0.8:0.1:0.1. The prediction accuracy was calculated from the average of five rounds of training and testing, which ensured the stability of the results and eliminated the effect of randomness. In each round, four subsets were utilized for the training set and one subset was allocated for the validation and testing sets. This approach was implemented once for each round until the entire cohort dataset, comprising 96 patients, was used as the testing set (*n* = 50) and validation set (*n* = 46). Data equalization and expansion were performed on the training and validation sets for both categories of patients (no data equalization or expansion were performed on the test set). The MedAugment algorithm was employed for data augmentation. This algorithm has been shown to be both efficient and effective in the context of automatic augmentation [26]. The framework used the following two transformation spaces: six pixel-level (photometric) transformations and eight spatial (geometric) transformations.

In this study, we employed the dense convolutional network (DenseNet) model, whose structure is depicted in Figure 3b [27]. The DenseNet architecture is predicated on a series of dense blocks, with each block comprising multiple convolutional layers. Each dense block receives the output of all preceding blocks, thereby establishing a dense pattern of connections between all the layers of the network. This configuration facilitates the efficient flow of information through the network. In selecting DenseNet121 as the backbone of the convolutional neural network, we considered the complexity of the data. Furthermore, in previous studies, we found that incorporating the squeeze-and-excitation block and spatial pyramid pooling layer into convolutional neural network models could markedly enhance the performance of deep learning models [28,29,30]. Consequently, we integrated them into the model. The structure of the squeeze-and-excitation block is illustrated in Figure 3b. The input convolutional layer was output after four operations. The squeeze-and-excitation block was applied to each convolutional block and its output was used as the input for the next convolutional block.

The pooling layer in the DenseNet output was replaced with the spatial pyramid pooling layer. The spatial pyramid pooling layer was implemented in three steps. First, the input feature maps were pooled using kernels of different sizes to obtain various output feature maps. Second, a fixed-size feature vector was obtained by merging the output feature maps of different sizes. Finally, the feature vectors were fed into the fully connected layer for classification, enabling the input of multiscale images that could then be processed and fed into a fixed-size fully connected layer. The multimodal image extracted 10,752 features following the spatial pyramid pooling layer. Notably, the images generated for this experiment were resized to 224 × 224 pixels utilizing the bilinear method for the interpolation of the images. The average size of the input ROIs was 54 × 48 pixels.

The more traditional deep residual network (ResNet)18 model and the Swin Transformer model were also used for training purposes [31,32,33]. The ResNet18 model is capable of capturing a greater range of features at varying depths within a dataset through the introduction of additional network layers. Concurrently, the ResNet18 model incorporates regularization to prevent gradient dispersion or explosion. The ResNet18 model was augmented with the squeeze-and-excitation block and spatial pyramid pooling layer. The squeeze-and-excitation block was employed as the output of each convolutional block and as the input of the subsequent convolutional layer. The spatial pyramid pooling layer replaced the global average pooling layer in the ResNet18 model and output the prediction results from its fully connected layer. The Swin Transformer model employs a hierarchical construction methodology and incorporates a sliding window mechanism, enabling information from multiple windows to be collected. Images undergo downsampling at 4, 8 and 16 times their original resolution as they pass through the patch partition block. This approach facilitates target classification and enables the model to process super-resolution images, focusing on both global and local information. The three-channel images were linearly transformed via the linear embedding layer in the model. The feature maps resulting from image chunking were doubled in depth and halved in width through the use of the patch merging layer.

### 2.6. Multimodal Fusion Artificial Intelligence Model Construction

**Figure 4:**
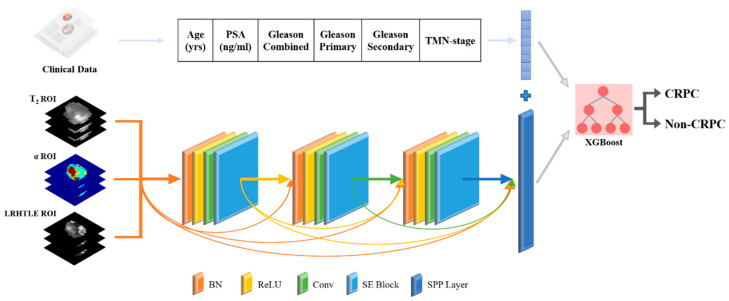
Dense-MFAI structure. The Dense-MFAI model comprises two components, SE-SPP-DenseNet and XGBoost. Images and clinical features are input into the multimodal fusion model and fused in the fully connected layer. PSA: prostate-specific antigen; XGBoost: eXtreme Gradient Boosting.

Following the construction of a pure deep learning classification model, a multimodal fusion artificial intelligence model that used deep learning and machine learning to integrate patient image data and baseline clinical feature information is proposed in this study. Consequently, the Dense-MFAI model was constructed. Baseline clinical information (age, baseline PSA, Gleason score and clinical TNM stage) and the imaging data of each patient were input into the model. The Dense-MFAI model is shown in Figure 4. Fusion learning across two data streams is complex and involves the construction of two separate machine learning channels. One channel is employed to accept the image features predicted by the deep learning model as the input, whereas the other channel is utilized to transform the clinical feature information into a one-dimensional numerical matrix as the input. The outputs of the clinical data and image feature channels are subsequently concatenated into a matrix input into the eXtreme Gradient Boosting classifier, which can then predict whether a patient could progress to CRPC within a median time of 12 months [34]. This study employed a two-stage training approach, wherein the deep learning model was initially trained and the eXtreme Gradient Boosting model was subsequently trained for classification. The data division across the two phases was identical. The eXtreme Gradient Boosting model is an optimized distributed gradient boosting machine learning algorithm designed to solve classification and regression problems efficiently, flexibly and portably. The inputs into the image feature channel are transformed from the fully connected layer of the deep learning model. The fully connected layer acts as a classifier in a convolutional neural network, which maps the trained distributed features into the sample labeling space, which is also a one-dimensional numerical matrix. The three deep learning models in this experiment were exclusively trained on the imaging data. The clinical data were extracted directly from the data tables and subsequently used for the training of the machine learning model. Additionally, the integration of image features and clinical features was performed by label correspondence. A more detailed study of the deep learning model architectures involved in this research can be found on GitHub (https://github.com/2271371/Dense-MFAI-model.git (accessed on 26 January 2025)).

### 2.7. Quantitative Assessment Metrics

In this study, a Kaplan–Meier curve was used to illustrate the progression of CRPC in patients and comparisons between groups were made using the log-rank test. The accuracy, sensitivity, specificity, positive predictive value (PPV), negative predictive value (NPV), receiver operating characteristic (ROC) curves and confusion matrices were calculated for the purpose of predicting patient progression to CRPC within 12 months for the MFAI model described above using the test set. Additionally, the area under the curve (AUC) was calculated.

## 3. Results

### 3.1. Patient Characteristics

Following data equalization and expansion, the image data consisted of 2454 images (originally 270) for patients with CRPC and 3161 images (originally 391) for patients without CRPC. The final training set comprised approximately 4900 images per round, with 2200 images from CRPC patients and 2700 images from non-CRPC patients. The validation set comprised approximately 690 images per round, with 270 images from CRPC patients and 420 images from non-CRPC patients. The test set comprised approximately 60 images per round, with 25 images from CRPC patients and 35 images from non-CRPC patients. It is noteworthy that the delineation of all subsets and datasets was performed at the patient level.

### 3.2. Parameter Settings for Each Model

Following the comparison of the outcomes of a series of preliminary experiments, the specific hyperparameters of the DenseNet, ResNet18 and Swin Transformer models were established, as indicated in Table 2. The same hyperparameters were used for each model in the five rounds of training. The hyperparameters were optimized based on the changes in accuracy and loss during training and validation. The grid search method was selected for the optimization process. The parameter combinations that performed the best in the validation set were ultimately selected. During the training process, if the loss using the validation set did not decrease after 10 epochs, the learning rate was adjusted to 0.5 times the initial learning rate. During the preliminary experiments, all three deep learning models converged before 50 epochs were reached when training. Consequently, the number of epochs was fixed at 50. Furthermore, an early stopping strategy was implemented, whereby training was terminated if the loss of the validation set did not decrease within 20 epochs to prevent overfitting. The stochastic gradient descent algorithm and the binary cross-entropy loss function were selected as the network optimizer and loss function, respectively. Additionally, during the training process, image enhancement was conducted, including the reversal of the vertical orientation of the images and normalization of the images by calculating the mean and variance for each set of images. Notably, image enhancement was only applied to the training images; the images used for testing were not enhanced.

### 3.3. Predictive Performance of MFAI Models

**Figure 5:**
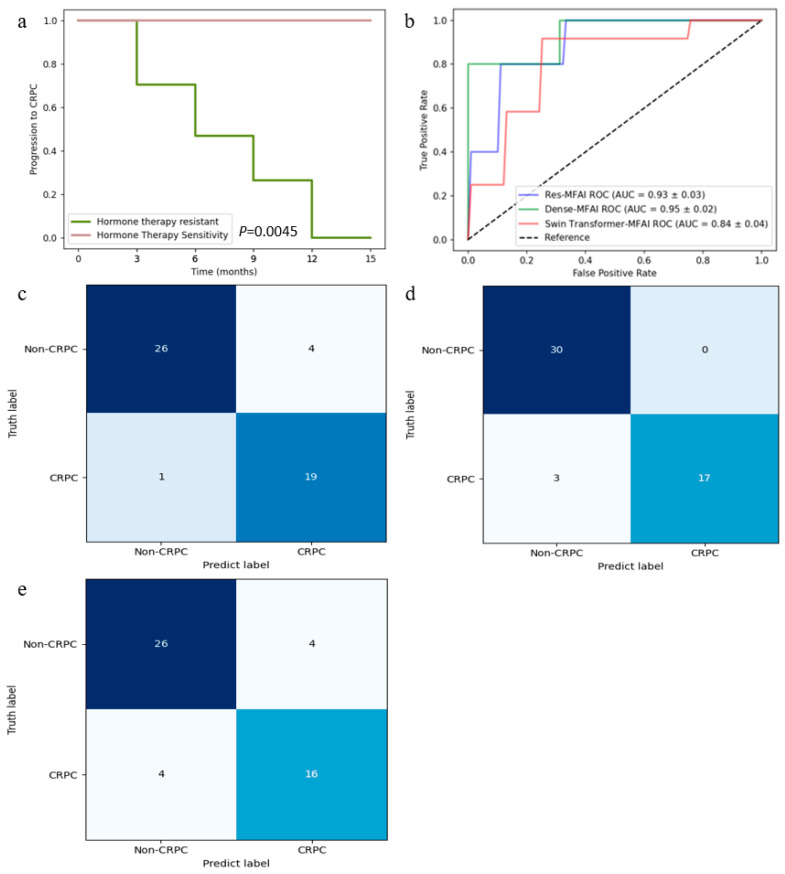
Performance evaluation of the MFAI models. (**a**) Kaplan–Meier curve for patients; (**b**) ROC curves for the prediction of CRPC via the Res-MFAI, Dense-MFAI and Swin Transformer-MFAI models; (**c**) confusion matrices for the Res-MFAI model; (**d**) confusion matrices for the Dense-MFAI model; (**e**) confusion matrices for the Swin Transformer-MFAI model.

This study proposes a multimodal fusion artificial intelligence detection framework based on deep learning and machine learning techniques to predict whether a PCa patient could progress to CRPC after 12 months of hormone therapy. This framework fused multimodal data with mpMRI parametric profiles, texture feature profiles and clinical feature information. The performance of three different MFAI models based on DenseNet, ResNet18 and the Swin Transformer was investigated. The receiver operating characteristic curves of the three models are shown in Figure 5b. The Dense-MFAI model achieved an accuracy of 94.2%, a sensitivity of 100.0%, a specificity of 82.1%, a positive predictive value of 89.6% and a negative predictive value of 100.0%. The Res-MFAI model achieved an accuracy of 92.7%, a sensitivity of 87.5%, a specificity of 96.4%, a positive predictive value of 97.5% and a negative predictive value of 84.1%. The Swin Transformer-MFAI model demonstrated an accuracy of 85.2%, a sensitivity of 87.8%, a specificity of 81.4%, a positive predictive value of 86.5% and a negative predictive value of 80.6%. The receiver operating characteristic curves of the aforementioned three models are presented in Figure 5b, where the AUCs of the Dense-MFAI, Res-MFAI and Swin Transformer-MFAI models were 0.945, 0.925 and 0.836, respectively. The confusion matrices to predict CRPC using the three models are shown in Figure 5c–e.

### 3.4. Effectiveness of Radiomics Feature Mapping at Predicting CRPC Progression

We also performed ablation experiments to validate the effectiveness of radiomics feature mapping at improving the prediction performance of the DenseNet, ResNet18 and Swin Transformer models. The results of the ablation experiments are shown in Table 3.

### 3.5. Importance of Baseline Clinical Features in Predicting CRPC Progression and Validation of Visualization

Patient multimodal images fused with clinical features were used as multimodal data for training and testing. However, current studies using artificial intelligence (AI) approaches to predict patient CRPC progression have only used imaging data. To illustrate the importance of clinical features in predicting CRPC progression, we conducted experiments using the three aforementioned pure deep learning methods for training and testing.

**Figure 6:**
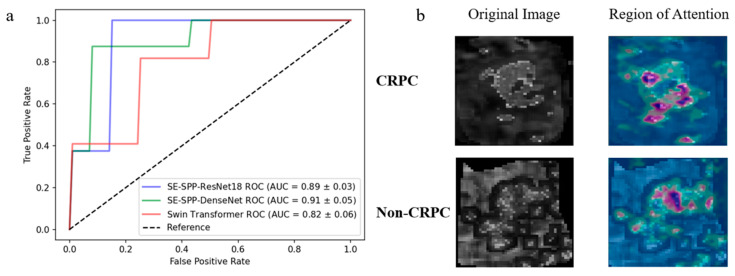
ROC curves and regions of attention. (**a**) ROC curves for CRPC prediction via the pure deep learning models SE-SPP-DenseNet, SE-SPP-ResNet18 and Swin Transformer; (**b**) predicted regions of attention using the SE-SPP-DenseNet model.

The AUCs of the SE-SPP-DenseNet, SE-SPP-ResNet18 and Swin Transformer models were 0.906, 0.896 and 0.807, respectively. The receiver operating characteristic curves and quantitative results of the three models are shown in Table 4 and Figure 6a, respectively.

In addition, with regard to the validation study, the region of attention for the SE-SPP-DenseNet model, which demonstrated the optimal prediction performance, was visualized (see Figure 6b). The utilization of distinct colors indicated how much each region contributed to the model: red and yellow (warm colors) indicated that the region contributed the most to the final classification decision and the model “pays the most attention” to these locations; green indicated moderate activation and the region had some influence on the judgment but was not critical; and blue (cool colors) indicated that the region contributed little to the output and the model was not sensitive to this part of the model. It was observed that the attention region was predominantly concentrated in the inner region of the tumor, while no activation was observed in the surrounding tissues of the tumor.

## 4. Discussion

In this study, we developed a multimodal fusion AI model to predict whether PCa patients could progress to CRPC after 12 months of hormone therapy. To our knowledge, no previous study has used deep learning and machine learning to apply imaging profile and clinical feature fusion to predict whether PCa patients could progress to CRPC. The findings demonstrated that the Dense-MFAI model exhibited the highest accuracy (94.2%) when predicting whether patients could progress to CRPC.

As illustrated in Figure 6a and Table 4, the prediction accuracy of DenseNet exceeded that of ResNet because the DenseNet modules were interconnected with each other, leading to the collection of information included from previous modules and the integration of information from subsequent modules. In the construction of an MFAI model, the selection of the deep learning model should be contingent upon the complexity of the data. In this study, the ResNet18 and DenseNet121 models were utilized instead of a more complex model. In addition to the potential for high model complexity, resulting in overfitting, an increase in the network complexity leads to a more complex solution space, hindering the identification of the optimal solution by the optimizer.

With limited arithmetic power, the accuracy of model prediction can be enhanced by focusing the computational weights on the information of the regions that are more critical to the current classification task. Therefore, we incorporated the squeeze-and-excitation block into the model. Furthermore, the spatial pyramid pooling layer not only enabled the model to adapt to different sizes of input data but also enhanced the classification accuracy by addressing issues of object distortion and spatial layout variations through multilevel pooling [30]. Consequently, the spatial pyramid pooling layer was also incorporated into the model in this study.

In this study, we combined parametric and radiomic feature mapping and jointly output them from the fully connected layer. As illustrated in Table 3, the incorporation of radiomic feature mapping into the model input resulted in a notable enhancement in the prediction outcomes. This finding demonstrates that multimodal image inputs could provide more effective information to the model [35]. Furthermore, the ablation experiments presented in Table 3 demonstrate that the prediction accuracy decreased when α-mapping was replaced with apparent diffusion coefficient mapping. The observed decline in accuracy could be attributed to the limitations of apparent diffusion coefficient mapping, which is susceptible to higher image noise and a lower spatial resolution.

As illustrated in Figure 5 and Table 4, the Dense-MFAI model exhibited the highest accuracy rate. The improved results could be attributed to the fact that the majority of the clinical information selected for this study was based on current clinical measures to determine whether a PCa patient has progressed to CRPC, as well as the assessment metrics of the prostate cancer risk classification developed by the National Comprehensive Cancer Network. Although it is essential to consider evolving patient data over time in clinical diagnoses, experimental evidence has demonstrated that the utilization of baseline clinical information can also provide valid insights to predict the progression of a patient to CRPC to a certain extent.

Compared with existing prediction models, we utilized a distinct prediction methodology and made further enhancements. Ali et al. [36] evaluated the performance of MR image radiomics analyses for the detection of prostate cancer and constructed a robust predictive model using a sophisticated hybrid descriptive inference approach. Zhou et al. [37] extracted 2553 texture features based on mpMRI parametric mapping via radiomics, resulting in an AUC of 0.768 for the prediction of patient progression to CRPC. In our study, the Dense-MFAI model was capable of extracting and learning features directly from raw image data, in contrast to machine learning models that utilize radiomics. This approach enabled the construction of an end-to-end model. In a study by Park et al. [6], a phased long short-term memory deep learning model was used for the prediction, with a final accuracy of 88.6%. In a study by Jin and Zhang et al. [38,39], the prediction of prostate cancer bone metastasis using clinical information, mpMRI and pathology images demonstrated that multimodal data can effectively improve prediction accuracy. In a study by Zhou et al. [37], a ResNet50 deep learning model combining radiomics and pathomics was used to predict the progression of CRPC in patients, with a final AUC of 0.86. The Dense-MFAI model proposed in this study employed patient clinical information in combination with imaging data, thereby enhancing the predictive performance in this specific dataset when compared with the other two methods and their respective datasets. Jun et al. [15] identified six gene signatures to predict the risk of patient progression. However, this method was unable to accurately identify patients. Furthermore, the study by Cairone et al. [40] demonstrated that physicians can be assisted in radiomics analyses by semi-automated segmentation methods. In a subsequent study, the semi-automatic segmentation method will be employed to enhance the annotation efficiency and alleviate the workload of physicians.

Our study had several limitations. First, as we performed a retrospective study, only patient cases within a four year period were selected as experimental data, which may have resulted in biased results. Second, due to the limitation with the sample size, this work was not validated with an independent test set. In the future, an external test set will be required to validate the work robustness of the model in a multi-institutional setting. Further research with larger datasets is needed to investigate the impact of the tumor location on the model’s performance and to explore how the structural information from different anatomical regions of the prostate influences quantitative imaging analyses. Third, the mpMRI equipment utilized in this study was identical, which reduced the variability of the data to some extent. Consequently, the results of this study require validation using a wider range of PCa patients and MRI from multiple vendors. Fourthly, the implementation of ROI delineation based on the sequence showing the maximum extent of cancer in this study resulted in a local volume effect. In future studies, we will perform systematic comparisons of different ROI delineation strategies (maximum, minimum or semi-automatic segmentation) to further optimize the accuracy and reproducibility of the model. Furthermore, subsequent studies could entail the ongoing development of regression models as a complement to existing classification models. This could furnish urologists with more comprehensive predictive information.

## 5. Conclusions

In this study, we proposed a multimodal fusion artificial intelligence model, Dense-MFAI, for the prediction of whether a PCa patient could progress to CRPC after 12 months of hormone therapy. This approach utilized the patient’s mpMRI parametric profile, radiomic feature mapping and baseline clinical features. The ablation experiments demonstrated that constructing a pure deep learning model via parametric mapping and radiomics feature mapping could enhance the classification performance of the original model. Moreover, the ablation experiments demonstrated that the fusion model constructed using baseline clinical features and imaging data exhibited an enhanced prediction ability compared with the pure deep learning model. The Dense-MFAI model accurately predicted whether PCa patients could progress to CRPC after 12 months of hormone therapy, with a final accuracy of 94.2% and an AUC of 0.945. We believe that urologists can use this model to assist with diagnoses to determine the most appropriate treatment plan and prognostic measures on the basis of whether a patient could progress to CRPC.

## Figures and Tables

**Table 1:** Magnetic resonance imaging parameters.

Imaging Sequence	FOV (mm)	Scan Matrix Size	TE (ms)	TR (ms)	Slice Thickness (mm)	Interval (mm)	Flip Angle (°)
T2WI	200	512 × 512	128	500	3	0.6	90
DWI	260	120 × 96	72	400	3	0.6	/
DCE	340	203 × 320	1.3	3.4	3.5	0	9

FOV: field of view; TE: echo time; TR: repetition time; T2WI: T2-weighted imaging; DWI: diffusion-weighted imaging; DCE: dynamic contrast-enhanced.

**Table 2:** Training parameters for the ResNet18, DenseNet and Swin Transformer models.

Parameter	Epoch	Learning Rate	Batch Size	Momentum	Weight Decay	Optimizer
ResNet18	50	0.001	48	0.9	0.0001	SGD
DenseNet	50	0.0001	48	0.9	0.0001	SGD
Swin Transformer	50	0.001	64	0.9	0.0001	SGD

SGD: stochastic gradient descent.

**Table 3:** Prediction results of a pure deep learning model for images using only mpMRI parameter mapping and after the fusion of radiomic feature mapping.

		Accuracy	Sensitivity	Specificity	PPV	NPV
SE-SPP-ResNet18	T2-ADC	76.3%	74.6%	80.3%	78.1%	75.7%
	T2-α	83.5%	80.3%	87.7%	87.3%	80.5%
	T2-ADC-α	85.2%	83.6%	87.0%	87.5%	83.6%
	T2-ADC-LRHGLE	80.4%	80.7%	86.4%	86.7%	81.8%
	T2-α-LRHGLE	90.3%	83.3%	100.0%	100.0%	81.6%
SE-SPP-DenseNet	T2-ADC	77.6%	80.5%	75.1%	77.2%	78.5%
	T2-α	85.1%	90.4%	75.2%	80.4%	91.2%
	T2-ADC-α	87.7%	89.6%	87.8%	88.9%	88.7%
	T2-ADC-LRHGLE	83.8%	82.4%	80.7%	81.2%	81.8%
	T2-α-LRHGLE	91.2%	91.9%	87.3%	91.7%	87.8%
Swin Transformer	T2-ADC	67.9%	75.4%	54.7%	62.2%	69.3%
	T2-α	73.1%	71.4%	75.4%	75.8%	70.5%
	T2-ADC-α	75.5%	82.4%	68.7%	70.3%	76.7%
	T2-ADC-LRHGLE	71.5%	79.7%	62.7%	69.9%	74.0%
	T2-α-LRHGLE	78.9%	98.1%	51.7%	73.3%	96.8%

**Table 4:** Results of predictions using baseline clinical features, unimodal images composed of mpMRI parameter maps and multimodal images fused with baseline clinical features.

	Accuracy	Sensitivity	Specificity	PPV	NPV
Baseline Clinical Features	70.4%	87.1%	41.6%	72.5%	67.7%
SE-SPP-ResNet18	90.3%	83.3%	100.0%	100.0%	81.6%
SE-SPP-ResNet18 + MFAI	92.7%	87.5%	96.4%	97.5%	84.1%
SE-SPP-DenseNet	91.2%	91.9%	87.3%	91.7%	87.8%
SE-SPP-DenseNet + MFAI	94.2%	100.0%	82.1%	89.6%	100.0%
Swin Transformer	78.9%	98.1%	51.7%	73.3%	96.8%
Swin Transformer + MFAI	85.2%	87.8%	81.4%	86.5%	80.6%

## Data Availability

Data generated or analyzed during the study are available from the corresponding author upon request.

## Abbreviations

PCa	Prostate cancer
CRPC	Castration-resistant prostate cancer
PSA	Prostate-specific antigen
mpMRI	Multiparametric magnetic resonance imaging
DCE-MRI	Dynamic contrast-enhanced MRI
MFAI	Multimodal fusion artificial intelligence
DWI	Diffusion-weighted imaging
ADC	Apparent diffusion coefficient
FOV	Field of view
TE	Echo time
TR	Repetition time
T2WI	T2-weighted imaging
PSE	Percentage signal enhancement
LRHGLE	Long-run high gray-level emphasis
ROI	Region of interest
SE block	Squeeze-and-excitation block
SPP layer	Spatial pyramid pooling layer
BN	Batch normalization
ReLU	Rectified linear units
Conv	Convolutional layer
GAP	Global average pooling
FC	Fully connected
DenseNet	Dense convolutional network
ResNet	Deep residual network
PPV	Positive predictive value
NPV	Negative predictive value
ROC	Receiver operating characteristic
AUC	Area under the curve
AI	Artificial intelligence
